# Protocol for FLOWMIST to profile autoreactivity of mouse monoclonal and serum antibodies by flow cytometry and microscopy

**DOI:** 10.1016/j.xpro.2026.104918

**Published:** 2026-10-28

**Authors:** Michael Ivanov, Uzair Ansari, Jan Wisniewski, Christian T. Mayer

**Affiliations:** 1 Experimental Immunology Branch, Center for Cancer Research, National Cancer Institute, National Institutes of Health, 10 Center Dr, Bethesda, MD 20892, USA

**Keywords:** Bioinformatics, Flow Cytometry, Immunology, Microscopy, Model Organisms, Antibody

## Abstract

Autoantibodies recognizing surface-bound or intracellular self-antigens are a hallmark of many autoimmune diseases. Here, we present a protocol for FLOWMIST to profile the autoreactivity of mouse monoclonal and serum antibodies using primary mouse splenocytes. We describe steps for antibody staining, quantitative flow cytometric screening, and confocal microscopy to classify antibody-binding patterns. Although demonstrated using mouse splenocytes, the workflow can be readily adapted to different target cells, antibody samples, species, and secondary detection reagents.

For complete details on the use and execution of this protocol, please refer to Simpson et al.^1^ and Newen et al.^2^

## Highlights

• Instructions for evaluating monoclonal antibodies and serum for autoreactivity
• Prepare samples for high-throughput screening via flow cytometry
• Confirm and characterize autoreactivity with confocal microscopy

## Abstract (editor highlights)

Publisher’s note: Undertaking any experimental protocol requires adherence to local institutional guidelines for laboratory safety and ethics.

## Graphical abstract

**Figure:**
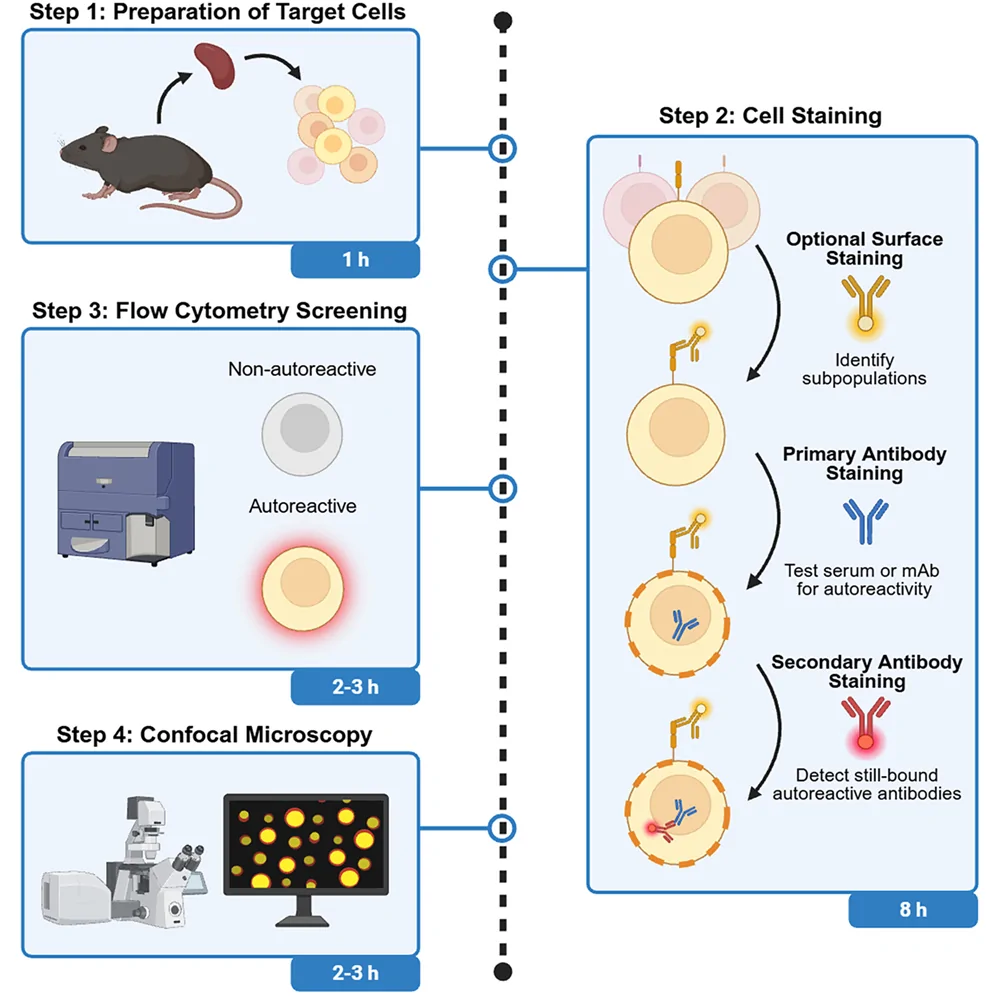

## Before you begin

**Timing: 2 h**

Autoantibodies recognizing self-antigens are a hallmark of many autoimmune diseases and are widely used to study immune tolerance, B cell selection, and disease pathogenesis. Existing approaches to evaluating autoantibodies include enzyme-linked immunosorbent assays (ELISAs) using purified self-antigens or cell extracts and indirect immunofluorescence using HEp-2 cells. ELISAs have the benefit of enabling high-throughput analysis, but they are limited to predefined antigens and do not preserve native cellular context or antibody binding patterns. Conversely, indirect immunofluorescence retains antigen organization within intact cells and reveals characteristic antibody binding patterns, but it is comparatively low throughput, relies on an immortalized human cell line, and may not fully represent the antigen repertoire of other species or primary cell populations.

Here, we present FLOWMIST (flow cytometry-based screening assay coupled with confocal microscopy or imaging flow cytometry), a workflow for profiling antibody binding using primary mouse splenocytes. Although this protocol uses primary mouse splenocytes, the target cells can be readily replaced with other primary cell populations or cell lines according to the experimental question. The protocol below describes the workflow for profiling mouse monoclonal antibodies and mouse serum antibodies.

1. Prepare 70% (v/v) Ethanol according to materials and equipment.

**Pause point:** 70% Ethanol can be prepared in advance and stored conservatively for up to 6 months at 20–25 °C.

2. Prepare FACS buffer.
  a. Prepare or thaw the 10% (w/v) bovine serum albumin (BSA) according to materials and equipment.
    ***Note:*** 40 mL aliquots can be stored at −20°C for up to 2 years and thawed as needed. Thawed aliquots can be stored at 4°C for up to 3 months.
  b. Prepare FACS buffer according to materials and equipment.
    ***Note:*** FACS buffer can be stored at 4°C for up to a month.
    **Pause point:** FACS buffer and BSA can be prepared in advance if reagents have been stored in the proper conditions and have not yet expired.
3. For each organ being collected, prepare 1 well of a 6-well plate.
  a. Fill the well with 6 mL of FACS buffer.
  b. Add a 70 μm cell strainer.

***Note:*** Keep the 6-well plate on ice.

4. Pre-label 15 mL tubes for each organ being collected and place at 4°C to pre-chill.
5. Prepare Foxp3 Fixation/Permeabilization working solution according to materials and equipment.

***Note:*** Foxp3 Fixation/Permeabilization working solution should be prepared fresh for each experiment. For 30 samples, 8 mL should be more than enough. Keep at 4°C or on ice when in use.

6. Prepare 1× BD Perm/Wash buffer according to materials and equipment.

***Note:*** 1× BD Perm/Wash buffer should be prepared fresh for each experiment. For 30 flow cytometry samples and 10 confocal samples, 75 mL should be more than enough. Keep at 4°C or on ice when in use.

### Innovation

FLOWMIST combines flow cytometry-based screening with confocal microscopy or imaging flow cytometry in a single workflow, enabling rapid screening of large numbers of antibody samples while preserving spatial information about antibody binding. Unlike conventional indirect immunofluorescence assays that rely on a single immortalized cell line, FLOWMIST allows investigators to select biologically relevant primary cells or cell lines according to the experimental question.

Autoreactivity testing of antibodies and serum has applications in determining cross-reactivity, evaluating molecular mimicry, and uncovering immune tolerance mechanisms. These purposes can involve different source organisms and antibody classes, necessitate screening against different antigen repertoires, and sometimes require looking at specific target populations. In these cases, it is beneficial to have a high-throughput method that is modular and can be adapted to many situations by exchanging target cells, antibody samples, secondary detection reagents, or imaging modalities without altering the overall experimental design.

### Institutional permissions

All animal procedures were approved by the National Cancer Institute Animal Care and Use Committee (NCI-ACUC; protocols #26-056 and EIB-114) and were performed in accordance with federal regulatory requirements and standards. The NIH Intramural Animal Care and Use Program is accredited by Association for Assessment and Accreditation of Laboratory Animal Care International (AAALAC International). Researchers performing this protocol should obtain approval from their local institutional animal care and use committee (or equivalent regulatory authority) and ensure that all experiments comply with applicable institutional and national guidelines.

### Preparation of splenocyte single-cell suspension

**Timing: 1 h (add 15–30 min for each additional mouse)**

**CRITICAL:** Perform all steps and keep all reagents on ice or at 4°C to minimize cell death.

***Note:*** While mouse splenocytes were used for the purposes of this experiment, be sure to choose appropriate target cells. One benefit of this workflow is that it allows for flexibility with the target cells used. If altering the target cells causes the antibody binding patterns to change, please see troubleshooting problem 1. Alternatively, if autoreactive positive-control antibodies are not providing an expected signal, see troubleshooting problem 2.

7. Euthanize one mouse and harvest the spleen.
  a. Euthanize one C57BL/6J mouse using CO_2_ inhalation followed by cervical dislocation as instructed by institutional guidelines.
  b. Liberally spray the mouse with 70% ethanol.
  c. Place the mouse on its back and make an incision through the upper left abdomen.
  d. Locate and remove the whole spleen.
  e. Collect the spleen in one well of a 6-well plate containing a 70 μm cell strainer and 6 mL of FACS buffer (recipe in materials and equipment).

***Note:*** One spleen is sufficient for approximately 30 samples. Depending on the number of samples, scale up the number of mice accordingly.

8. Prepare a single-cell suspension of the spleen.
  a. Using the plunger of a 3 mL syringe, gently press the spleen through the cell strainer until only connective tissue remains.
  b. Flush the suspension through the cell strainer three times using a 10 mL serological pipette.
  c. Transfer the solution to a pre-chilled 15 mL tube.
  d. Wash the cell strainer again with an additional 6 mL of FACS buffer, passing the buffer through the strainer three times.
  e. Transfer the solution to the same 15 mL tube.
9. Count the cells.
  a. Centrifuge the single-cell suspension for 7 min at 396 × *g* at 4°C. Aspirate the supernatant.
  b. Resuspend the pellet in 1 mL of ACK lysing buffer and incubate on ice for 1 min.
  c. Add 9 mL of FACS buffer and mix by inverting the tube.
  d. Dilute the cell suspension for counting.
    i. Transfer 10 μL of the cell suspension to a well of a 96-well plate containing 40 μL of FACS buffer.
    ii. Mix by pipetting 10 times.
    iii. Transfer 10 μL of the dilution to a second well containing 40 μL of FACS buffer.
    iv. Mix by pipetting 10 times.
    v. Transfer 10 μL of the twice-diluted sample to a third well.
    vi. Add 10 μL of 0.4% trypan blue.
    vii. Mix by pipetting 10 times and take 10 μL for counting (final dilution factor: 50).
  e. Load the 10 μl of diluted and stained sample into one side of a Neubauer chamber beneath the coverslip.
    **CRITICAL:** Ensure that the chamber fills evenly beneath the coverslip without introducing air bubbles.
  f. Under a microscope, count cells in the four large squares of the grid, excluding cells on the lower and right outside borders.
  g. Calculate the total cell number:
    $$Total\;cells=\left(\frac{Cells\;counted}{Large\;squares}\right)\times \left(Dilution\;factor\right)\times 10,000\times \left(Suspension\;volume\;\left(mL\right)\right)$$
  h. While counting, centrifuge the splenocyte suspension for 7 min at 396 × g at 4°C. Aspirate the supernatant.
    ***Note:*** An automated cell counter may be used instead of a Neubauer chamber.
10. Add the cells to a 96-well plate.
  a. Resuspend the spleen pellet to 1 × 10^7^ cells/mL in FACS buffer. Remove any visible fat or connective tissue using a pipette tip.
  b. Add 200 μL, or 2 million cells, to each well of a round-bottom 96-well plate.
  c. Centrifuge the plate for 3 min at 396 × *g* at 4°C.
  d. Flick the plate over the sink to discard the supernatant. Dry the top of the plate with a paper towel.

**CRITICAL:** Resuspend the cell pellet completely before aliquoting into the 96-well plate and periodically during sample loading to ensure equal cell numbers between wells.

## Key resources table

**Table:** 

REAGENT or RESOURCE	SOURCE	IDENTIFIER
**Antibodies**		

BD Pharmingen™ PE Hamster Anti-Mouse CD3e (1:32 dilution)	BD Bioscience	Cat#553064; RRID:AB_394597
Alexa Fluor® 647 AffiniPure® F(ab')_2_ Fragment Goat Anti-Human IgG, Fcγ fragment specific (1 μg/mL)	Jackson ImmunoResearch	Cat#109-606-098; RRID:AB_2337899
Alexa Fluor® 647 AffiniPure® F(ab')_2_ Fragment Goat Anti-Mouse IgG, Fcγ fragment specific (1 μg/mL)	Jackson ImmunoResearch	Cat# 115-606-071; RRID:AB_2338927
673/719 monoclonal antibody (autoreactive control) (ten-fold dilutions from 10 μg/mL to 0.01 μg/mL)	In-house	Mayer et al.^3^
ED38 monoclonal antibody (polyreactive control) (ten-fold dilutions from 10 μg/mL to 0.01 μg/mL)	In-house	Wardemann et al.^4^; Simpson et al.^1^
mGO53 monoclonal antibody (negative control) (ten-fold dilutions from 10 μg/mL to 0.01 μg/mL)	In-house	Wardemann et al.^4^; Simpson et al.^1^
Fc Block, CD16/CD32, Clone 2.4G2 (1:20 dilution)	In-house	N/A
FITC anti-mouse CD19 Antibody (1:10 dilution)	BioLegend	Cat#101506; RRID:AB_312825

**Biological samples**		

Serum from Bcl2^Early^Aicda^−/−^ mice (negative control) (four-fold dilutions from 1:640 to 1:40,960) (Mouse: Mb1^Cre^Rosa26^LSL−Bcl2^Aicda^−/−^, 36 weeks old, female)	In-house	Newen et al.^2^
Serum from Bcl2^Early^ mice (autoreactive control) (four-fold dilutions from 1:640 to 1:40,960) (Mouse: Mb1^Cre^Rosa26^LSL−Bcl2^, 36 weeks old, female)	In-house	Newen et al.^2^
Serum from C57BL/6J mice (negative control) (four-fold dilutions from 1:640 to 1:40,960) (Mouse: C57BL/6J, wild type, 36 weeks old, female)	In-house	Newen et al.^2^

**Chemicals, peptides, and recombinant proteins**		

ACK Lysing Buffer	KD Medical	Cat#RGC-3015
DPBS (10X), no calcium, no magnesium	ThermoFisher Scientific	Cat#14200075
DPBS, no calcium, no magnesium	ThermoFisher Scientific	Cat#14190144
DAPI	Sigma-Aldrich	Cat#D9542
Trypan Blue Stain 0.4%	LONZA	Cat#17-942E
Bovine Serum Albumin - Fraction V (Immunoglobulin and Protease Free)	Rockland Immunochemicals	Cat#BSA-1000
UltraPure™ 0.5M EDTA, pH 8.0	ThermoFisher Scientific	Cat#15575020
Ethyl Alcohol, 200 Proof	Warner Graham	CAS#64-17-5

**Critical commercial assays**		

LIVE/DEAD™ Fixable Aqua Dead Cell Stain Kit, for 405 nm excitation	ThermoFisher Scientific	Cat#L34966
eBioscience™ Foxp3/Transcription Factor Staining Buffer Set	ThermoFisher Scientific	Cat#00-5523-00
BD Perm/Wash™ Buffer	BD Bioscience	Cat#554723

**Experimental models: Organisms/strains**		

Mouse: C57BL/6J (wild type, aged 2 months, male)	Jackson Laboratory	RRID:IMSR_JAX:000664

**Software and algorithms**		

Custom ImageJ macro	This study	https://github.com/janwisn/Splenocytes
FlowJo_V10.10.1	Waters Corp	N/A
INSPIRE v200.1.681.0	Cytek	N/A
ImageJ	National Institutes of Health (NIH)	N/A
NIS-Elements AR version 5.02.01	Nikon	N/A

**Other**		

BD LSRFortessa X-20 Cell Analyzer	BD Bioscience	N/A
Corning® Falcon® 70 μm Cell Strainer	Millipore Sigma	Cat#CLS352350
Nylon Membrane Filter, 60.0 μm Pore Size	Millipore Sigma	Cat#NY6000010
BD Luer-Lok™ Syringe	BD	Cat#309657
μ-Dish 35 mm, high	Ibidi	Cat#81156
Corning® 96-well Clear Round Bottom Polystyrene Not Treated Microplate	Corning	Cat#3788
Falcon® 6-well Clear Flat Bottom TC-treated Multiwell Cell Culture Plate	Corning	Cat#353046
Corning® 15 mL PP Centrifuge Tubes	Corning	Cat#430766
Nikon Ti2/Yokogawa CSU-W1 spinning disk confocal microscope	Nikon	N/A
10 mL Stripette™ Serological Pipets, Polystyrene, Individually Paper/Plastic Wrapped, Sterile	Corning	Cat#4488
S1 Pipet Fillers	ThermoFisher Scientific	Cat#9541
Sorvall Legend XTR Centrifuge	ThermoFisher Scientific	Cat#75004521
Double Neubauer Counting Chamber	Hausser Scientific	Cat#3100
Zeiss Axio Vert.A1 Microscope	Zeiss	Cat#431030-9040-000
Falcon® 5 mL Round Bottom Polystyrene Test Tube, without Cap, Nonsterile	Corning	Cat#352008
Hamamatsu ORCA-Flash4.0 camera	Hamamatsu	Cat#C13440
Nalgene™ Rapid-Flow™ Sterile Disposable Filter Unit, 0.2 μm, SFCA, 50 mm, 250 mL	ThermoFisher Scientific	Cat#157-0020
Eppendorf™ Safe-Lock 1.5 mL Microtube	Eppendorf	Cat#05-402-27
CFI Plan Apochromat Lambda D 60× Oil Objective	Nikon	Cat#MRD71670

## Materials and equipment

**Table:** FACS Buffer

Reagent	Final concentration	Amount
10X DPBS (without calcium or magnesium)	1×	50 mL
0.5 M EDTA	2 mM	2 mL
10% w/v BSA	0.1% w/v	5 mL
diH_2_O	N/A	443 mL
**Total**	**N/A**	500 mL

Store at 4°C for up to 1 month.

### 70% (v/v) ethanol

• Mix 7 parts of 200 proof ethyl alcohol with 3 parts of diH_2_O.
• 100 mL in a spray bottle is plenty. To prepare, mix 70 mL of ethanol with 30 mL of diH_2_O.
• Store at 20–25°C for up to 6 months.

### Foxp3 fixation/permeabilization working solution

• Mix 1 part of 4X Foxp3 Fixation/Permeabilization concentrate with 3 parts of Foxp3 Fixation/permeabilization diluent.
• For 30 samples, 8 mL of solution should be more than enough. To make 8 mL, mix 2 mL of Foxp3 Fixation/Permeabilization concentrate with 6 mL of Foxp3 Fixation/permeabilization diluent.
• Make the solution fresh and keep it at 4°C or on ice during the experiment.

### BD Perm/wash buffer

• If crystals are present in the 10X BD Perm/Wash buffer, dissolve them first by placing the bottle in a 37°C water bath for 10 minutes.
• Mix 1 part of the 10X BD Perm/Wash buffer with 9 parts of diH_2_O.
• For 30 flow cytometry and 10 confocal microscopy samples, 75 mL of buffer should be more than enough. To make 75 mL, mix 7.5 mL of 10X BD Perm/Wash buffer with 67.5 mL of diH_2_O.
• Make the solution fresh and keep it at 4°C or on ice during the experiment.

### 10% (w/v) bovine serum albumin

• Add approximately 150 mL of diH_2_O to 20 mL of 10X DPBS.
• Add 20 g of BSA while gently swirling to disperse the powder.
• Bring the final volume to 200 mL with diH_2_O and mix until the BSA is completely dissolved.
• Sterile filter the solution through a 0.2 μm filter.
• Aliquot into 40 mL portions and store at −20°C for up to 2 years.
• Thaw aliquots as needed and store at 4°C for up to 3 months.

## Step-by-step method details

### Viability staining

**Timing: 1.25 h**

Staining the cells with a fixable dead cell marker allows for gating on cells that were alive at the time of fixation. This helps eliminate noise from autofluorescent dead cells.

**CRITICAL:** Unless otherwise specified in this protocol, perform all steps and keep all reagents either on ice or at 4°C.

1. Wash the cells three times in DPBS.
  a. Resuspend the cells in 200 μL of DPBS.
  b. Centrifuge the plate for 3 min at 396 × *g* at 4°C.
  c. Flick the plate over the sink to discard the supernatant. Dry the top of the plate with a paper towel.
  d. Repeat steps 1a-c two more times.

***Note:*** This protocol contains many washing steps. Ensure good technique is employed when discarding the supernatant and resuspending the pellet to avoid cell loss (troubleshooting problem 3).

2. Add 50 μL of DPBS containing LIVE/DEAD Fixable Aqua Dead Cell Stain (1:500).

***Note:*** Depending on the lasers and channels available on the flow cytometer, other fixable dead cell markers may be used. Ensure that whichever stain is used allows for later DAPI staining and does not interfere with imaging.

***Note:*** For compensation purposes, include an unstained sample that receives only DPBS.

3. Incubate at 4°C in the dark for 30 min.

***Note:*** This is a suitable time to prepare the Fc block solution used in step 5.

4. Wash the cells twice in FACS buffer.
  a. Add 200 μL of FACS buffer directly to the wells.
  b. Centrifuge the plate for 3 min at 396 × *g* at 4°C.
  c. Flick the plate over the sink to discard the supernatant. Dry the top of the plate with a paper towel.
  d. Resuspend the cells in 200 μL of FACS buffer.
  e. Repeat steps 4b-c one more time.

### Fc receptor blocking

**Timing: 0.5 h**

Fc blocking prevents Fc receptors on the target cells from nonspecifically binding the primary and secondary antibodies used in subsequent steps.

***Note:*** If high background staining is detected in non-reactive samples used as negative controls, see troubleshooting problem 4.

5. Resuspend the cells in 50 μL of FACS buffer containing Fc block (clone 2.4G2, 1:20).

***Note:*** The Fc block used in this protocol (clone 2.4G2) is produced in-house. Commercial Fc blocking reagents may be substituted according to the manufacturer's recommended concentration.

***Note:*** When profiling mouse serum antibodies using mouse splenocytes, the anti-mouse IgG secondary antibody may cross-react with the rat IgG Fc block clone 2.4G2, potentially contributing to background staining. Users concerned about this potential source of background may omit Fc block or substitute an alternative blocking reagent that does not react with the secondary antibody.

6. Incubate on ice for 15 min.

***Note:*** This is a suitable time to prepare the surface staining solutions used in step 8.

7. Wash the cells once in FACS buffer.
  a. Add 200 μL of FACS buffer directly to the wells.
  b. Centrifuge the plate for 3 min at 396 × g at 4°C.
  c. Flick the plate over the sink to discard the supernatant. Dry the top of the plate with a paper towel.

### Surface staining

**Timing: 1.5 h**

Surface staining enables antibody autoreactivity to be quantified within defined target cell populations.

8. Resuspend the cells in 50 μL of FACS buffer containing FITC anti-mouse CD19 (1:10) and PE anti-mouse CD3ε (1:32) antibodies. For controls, resuspend cells in 50 μL of FACS buffer only.

***Note:*** For compensation purposes, include unstained and live/dead single-stain samples that receive only FACS buffer, as well as single stains that receive only one of each antibody. To create a single stain for the secondary antibody, have a sample that receives FACS buffer instead of surface antibodies, is subsequently stained with a positive-control primary antibody, and finally receives the corresponding secondary antibody.

**CRITICAL:** Surface antibodies must be selected carefully to ensure they are not recognized by the secondary antibody used for autoreactivity detection. We routinely use FITC anti-mouse CD19 (mouse IgA) and PE anti-mouse CD3ε (Armenian hamster IgG), which are compatible with the anti-human IgG secondary antibody used for monoclonal antibody profiling. Alternative surface antibodies may be used but should first be tested for compatibility with the selected secondary antibody. Antigen expression can vary depending on cell populations. See troubleshooting problem 1 if altering target cell populations is affecting the antibody staining patterns. If autoreactive positive-control antibodies are not providing an expected signal in the target cells, see troubleshooting problem 2.

**CRITICAL:** Surface staining was omitted for mouse serum profiling because the anti-mouse IgG secondary antibody can also recognize surface-staining antibodies. To combine serum profiling with surface staining, use surface and secondary antibodies that have been verified to be compatible.

***Note:*** If cell loss is a concern, omitting surface staining may help in reducing the number of washes (troubleshooting problem 3).

9. Incubate for 45 min at 4°C in the dark.

***Note:*** This is a suitable time to prepare the dilutions of primary antibodies for step 16. If more time is needed, dilutions can also be made during the fixation in step 13.

10. Add DPBS to the cells and pellet them.
  a. Add 200 μL of DPBS buffer directly to the wells.
  b. Centrifuge the plate for 3 min at 396 × g at 4°C.
  c. Flick the plate over the sink to discard the supernatant. Dry the top of the plate with a paper towel.
11. Wash the cells two times in DPBS.
  a. Resuspend the cells in 200 μL of DPBS.
  b. Centrifuge the plate for 3 min at 396 × *g* at 4°C.
  c. Flick the plate over the sink to discard the supernatant. Dry the top of the plate with a paper towel.
  d. Repeat steps 11a-c one more time.

### Fixation and permeabilization

**Timing: 1 h**

Fixing and permeabilizing the cells allows the antibodies of interest to interact with intracellular antigens.

***Note:*** To assess autoreactivity against surface antigens by flow cytometry, omit fixation and permeabilization and perform primary and secondary antibody staining and washes in FACS buffer.

***Note:*** If the fixation process affects the binding of antibodies, see troubleshooting problem 2 and troubleshooting problem 5.

12. Resuspend the cells in 200 μL of Foxp3 Fixation/Permeabilization working solution.
13. Incubate for 30 min at 4°C in the dark.
14. Remove the fixation/permeabilization solution.
  a. Centrifuge the plate for 3 min at 396 × *g* at 25°C.
  b. Flick the plate over the sink to discard the supernatant. Dry the top of the plate with a paper towel.

***Note:*** At this point you may no longer see a pellet after spinning. This is an expected result of fixation/permeabilization and not necessarily indicative of cell loss.

15. Wash the cells two times in BD Perm/Wash buffer.
  a. Resuspend the cells in 200 μL of BD Perm/Wash buffer.
  b. Centrifuge the plate for 3 min at 396 × *g* at 25°C.
  c. Flick the plate over the sink to discard the supernatant. Dry the top of the plate with a paper towel.
  d. Repeat Steps 15a-c one more time.

**CRITICAL:** We avoided eBioscience Permeabilization Buffer when staining CD19, as it seems to alter FITC anti-mouse CD19 staining signal.

***Note:*** Be gentle when resuspending samples in BD Perm/Wash buffer; do not over-pipette or pipette too harshly, as this may cause cell lysis (troubleshooting problem 3).

### Primary antibody staining

**Timing: 2 h**

Monoclonal or serum antibodies bind to target cells if they recognize cellular antigens.

16. Resuspend the cells in 50 μL of BD Perm/Wash buffer containing monoclonal antibodies or serum. In the case of unstained and single-stain samples, resuspend in 50 μL of BD Perm/Wash buffer only.

***Note:*** For initial screening, we recommend testing monoclonal antibodies at 100 μg/mL and serum at a 1:640 dilution. Positive samples may subsequently be titrated to estimate relative antibody binding or determine endpoint titers. In this protocol, representative control monoclonal antibodies were analyzed using ten-fold serial dilutions from 10 to 0.01 μg/mL, whereas serum samples were analyzed by four-fold serial dilution beginning at 1:640 and ending at 1:40,960.

***Note:*** Include positive and negative control monoclonal antibodies or serum samples in every experiment. A good control to include is a sample which receives all staining steps except for the primary antibody (troubleshooting problem 4). This allows for the background signal of the secondary antibody to be evaluated.

***Note:*** If the serum contains a visible precipitate, centrifuge the sample for 5 minutes at 16,200 × *g* to help clear debris. If the pellet of debris is not disturbed, the serum sample should be sufficiently clarified to use.

***Note:*** Problems can arise from using primary antibodies at suboptimal concentrations including altered binding between target cells (troubleshooting problem 1), false negatives (troubleshooting problem 2), false positives (troubleshooting problem 4), inconsistent signals (troubleshooting problem 6), and variations between flow cytometry and confocal imaging (troubleshooting problem 7). It is recommended to titrate primary antibodies.

17. Incubate for 1 h at 4°C.

***Note:*** This is a suitable time to prepare secondary antibody solutions used in step 20.

18. Add BD Perm/Wash buffer to the cells and pellet them.
  a. Add 200 μL of BD Perm/Wash buffer directly to the wells.
  b. Centrifuge the plate for 3 min at 396 × *g* at 25°C.
  c. Flick the plate over the sink to discard the supernatant. Dry the top of the plate with a paper towel.
19. Wash the cells three times in BD Perm/Wash buffer.
  a. Resuspend the cells in 200 μL of BD Perm/Wash buffer.
  b. Incubate for 5 min at 20–25°C protected from light.
  c. Centrifuge the plate for 3 min at 396 × *g* at 25°C.
  d. Flick the plate over the sink to discard the supernatant. Dry the top of the plate with a paper towel.
  e. Repeat Steps 19a-d two more times.

***Note:*** If high background fluorescence is observed in negative control samples, consider increasing the number or volume of washes (troubleshooting problem 4).

### Secondary antibody staining

**Timing: 2 h**

Fluorophore-conjugated secondary antibodies bind to primary antibodies that recognize cellular antigens and enable detection by flow cytometry, confocal microscopy, or imaging flow cytometry.

***Note:*** If non-reactive primary antibodies used as a negative control are showing high background fluorescence, see troubleshooting problem 4.

20. Resuspend the cells in 50 μL of BD Perm/Wash buffer containing 1 μg/mL of the appropriate secondary antibody. For unstained and single-stain controls, resuspend cells in 50 μL of BD Perm/Wash buffer only.

***Note:*** Our monoclonal antibodies containing human IgG constant regions are detected with an anti-human secondary antibody conjugated to Alexa Fluor 647. Mouse serum IgG is detected with an anti-mouse secondary antibody also conjugated to Alexa Fluor 647. Alternative secondary antibodies may be used, but they should first be titrated and tested for cross-reactivity with surface-staining antibodies or endogenous immunoglobulins expressed by the target cells (e.g., mouse IgM/IgD on B cells).

***Note:*** Secondary antibodies that have not been titrated or checked for compatibility with primary and surface antibodies can introduce several problems. This can lead to altered binding patterns in target cells (troubleshooting problem 1), false negatives (troubleshooting problem 2), false positives (troubleshooting problem 4), and inconsistent signals (troubleshooting problem 6). Make sure that the secondary antibody recognizes the species and isotype of primary antibodies and does not cross-react with the surface stain antibodies. It is recommended to titrate secondary antibodies.

21. Incubate at 4°C in the dark for 1 h.
22. Add BD Perm/Wash buffer to the cells and pellet them.
  a. Directly add 200 μL of BD Perm/Wash buffer to the wells.
  b. Centrifuge the plate for 3 min at 396 × *g* at 25°C.
  c. Flick the plate over the sink to discard the supernatant. Dry the top of the plate with a paper towel.
23. Wash the cells three times in BD Perm/Wash buffer.
  a. Resuspend the cells in 200 μL of BD Perm/Wash buffer.
  b. Incubate the plate for 5 min at 20–25°C in the dark.
  c. Centrifuge the plate for 3 min at 396 × *g* at 25°C.
  d. Flick the plate over the sink to discard the supernatant. Dry the top of the plate with a paper towel.
  e. Repeat Steps 23a-d two more times.

***Note:*** If high background fluorescence is observed in negative control samples, consider increasing the number or volume of washes (troubleshooting problem 4).

24. Resuspend the cells in 100 μL of FACS buffer and take half for flow cytometry. Leave the remaining cells in the dark at 4°C for downstream microscopy.

***Optional:*** Single-stain flow cytometry samples may be diluted up to 200 μl with FACS buffer to allow for more time to compensate samples.

***Note:*** If cell number is a concern, consider acquiring the full sample and performing imaging in an independent experiment (troubleshooting problem 3).

**Pause point:** The stained cells can be stored for up to 4 days in the dark at 4°C before acquiring them via flow cytometry without noticeable loss of signal. If modifications to the protocol are introduced, we recommend acquiring samples immediately or first confirming signal stability under the modified conditions.

### Flow cytometry

**Timing: 2 h for 30 samples**

Flow cytometry enables quantitative, high-throughput screening for antibody autoreactivity based on Alexa Fluor 647 secondary antibody fluorescence. Positive samples can subsequently be analyzed by confocal microscopy or imaging flow cytometry to classify antibody binding patterns.

25. Filter the cells through a 60 μm nylon mesh into 5 mL FACS tubes without lids. Keep them at 20–25°C but protect the tubes from light.
26. Set the voltages and compensation of the flow cytometer according to the unstained and single-stained control cells.
27. Acquire the full tube for each sample.

**CRITICAL:** Wipe the sample injection needle and let buffer drip from the needle into a waste container after every sample to minimize cross-contamination between samples.

***Note:*** In our experience, around 50,000 cells are acquired with this flow cytometry workflow, though 10,000 should be sufficient for analysis if time or cells are limited.

28. Export the FCS files and analyze them with FlowJo (Figure 1).
  a. Gate the cells using FSC/SSC, followed by singlets, and live cells.
    ***Optional:*** If desired, further gate target cell populations (e.g., B cells or T cells).
  b. Determine the Alexa Fluor 647 MFI.
  c. Calculate the MFI ratio as:
    $$MFI\;Ratio=\frac{{MFI}_{Sample}}{{MFI}_{Negative\;Control}}$$
    ***Note:*** In our studies^1^^,^^2^ and this protocol, samples with an MFI ratio >3 were considered positive for autoreactivity. Users may adjust this threshold depending on the experimental system and appropriate positive and negative controls.
    ***Note:*** If possible, include the same positive and negative control antibodies or serum samples in every experiment to facilitate comparisons between experiments. We further recommend starting each experiment using the same flow cytometer settings, only adjusting photomultiplier tube voltages and compensation based on the unstained and single-stained controls when necessary. If serum samples are providing weak or inconsistent signals, see troubleshooting problem 6.

**Figure 1:**
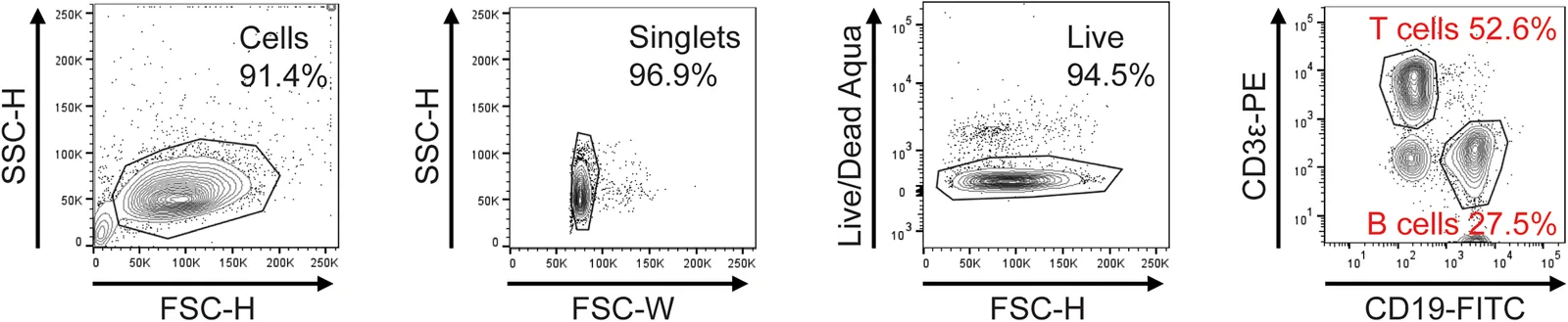
Flow cytometry gating scheme Representative flow cytometry gating scheme of monoclonal antibodies. The same scheme was applied to samples stained with serum, though without the B and T cell gating due to the omission of surface stains.

### DAPI staining

**Timing: 0.5 h**

DAPI staining provides a nuclear reference for confocal microscopy or imaging flow cytometry and facilitates classification of antibody binding patterns.

29. For autoreactive samples of interest, take the remaining half of the sample stored in step 24 along with the unstained and single-stained controls and transfer them to a new 96 well plate.

***Note:*** In our previous study,^1^ samples for confocal microscopy were prepared independently. This may be useful if there are restraints with machine availability, timing, or cell counts (troubleshooting problem 3). Here, we instead recommend using the remaining sample from flow cytometry to reduce sample preparation time.

30. Remove the FACS buffer.
  a. Centrifuge the plate for 3 min at 396 × *g* at 25°C.
  b. Flick the plate over the sink to discard the supernatant. Dry the top of the plate with a paper towel.
31. Wash the cells three times in BD Perm/Wash buffer.
  a. Resuspend the cells in 200 μL of BD Perm/Wash buffer.
  b. Centrifuge the plate for 3 min at 396 × *g* at 25°C.
  c. Flick the plate over the sink to discard the supernatant. Dry the top of the plate with a paper towel.
  d. Repeat Steps 31a-c two more times.
32. Stain the cells with DAPI.
  a. Resuspend the experimental samples and the LIVE/DEAD Aqua single-stained control in 50 μL of BD Perm/Wash Buffer containing 2 μg/mL of DAPI.
  b. Resuspend the unstained control and the remaining single-stained controls in 50 μL of BD Perm/Wash Buffer without DAPI.
  c. Incubate the plate for 5 min at 20–25°C in the dark.

***Note:*** For simplicity, the LIVE/DEAD Aqua single-stained control prepared for flow cytometry is subsequently stained with DAPI and used as the DAPI single-stained control for confocal microscopy. DAPI stains the nuclei of all cells, including live cells, allowing the DAPI channel to be optimized using this sample. Any residual LIVE/DEAD Aqua signal does not interfere with identification of DAPI-stained nuclei under the imaging conditions used here. Alternatively, an additional unstained sample may be prepared during flow cytometry and subsequently stained with DAPI to generate a dedicated DAPI single-stained control.

33. Add BD Perm/Wash buffer to the cells and pellet them.
  a. Directly add 200 μL of BD Perm/Wash buffer to the wells.
  b. Centrifuge the plate for 3 min at 396 × *g* at 25°C.
  c. Flick the plate over the sink to discard the supernatant. Dry the top of the plate with a paper towel.
34. Wash the cells two times in BD Perm/Wash buffer.
  a. Resuspend the cells in 200 μL of BD Perm/Wash buffer.
  b. Incubate the plate for 5 min at 20–25°C in the dark.
  c. Centrifuge the plate for 3 min at 396 × *g* at 25°C.
  d. Flick the plate over the sink to discard the supernatant. Dry the top of the plate with a paper towel.
  e. Repeat Steps 34a-d one more time.

### Preparation of imaging dish

**Timing: 0.5 h**

Transferring the cells from the 96 well plate to the imaging dish allows for confocal microscopy images to be taken of the samples. Proper preparation allows cells to adhere to the dish.

***Note:*** Prepare the imaging dish immediately before imaging.

35. Resuspend each cell pellet in 50 μL of DPBS, then mix the cell suspension with 350 μL of DPBS in a 1.5 mL microcentrifuge tube.
36. Transfer each sample to a separate μ-Dish.
  a. On flat surface, add 400 μL of the cell suspension on the inner well of the μ-Dish surface.
  b. Gently tap the dish to ensure the suspension evenly covers the inner well.
  c. Close the lid completely.
37. Incubate the μ-Dishes for 10 min at 20–25°C, protected from light, to allow the cells to settle and adhere.

***Note:*** The 10 min settling time described here is optimized for mouse splenocytes and may require optimization for other primary cell types or cell lines.

38. Remove the non-adherent cells from the μ-Dishes one at a time.
  a. Tilt the μ-Dish.
  b. Carefully aspirate the DPBS from the bottom edge of the inner well.
    **CRITICAL:** Don’t touch the imaging surface, avoid exposing the cells to air longer than necessary.
  c. Immediately move on to step 39 before beginning processing of the next μ-Dish.
39. Add 400 μL of fresh DPBS along the side wall of the inner well to cover the cells, then repeat steps 38–39 with the next μ-Dish.

**CRITICAL:** Add fresh DPBS slowly along the side of the inner well to avoid detaching adhered cells.

### Confocal image acquisition

**Timing: 1.5 h for 10 samples (timing depends on the number of samples and fields acquired)**

Properly setting up the confocal microscope is essential to accurately analyze the samples, distinguishing non-reactive from autoreactive samples, as well as identifying binding patterns in later steps. See Table 1 for the fluorophores used, with their corresponding staining targets, excitation wavelengths, and detection ranges used in this experiment.

***Note:*** See troubleshooting problem 7 if the flow cytometry and confocal imaging do not match. This may be a result of suboptimal antibody dilutions or microscope settings.

***Note:*** We acquired images using a Nikon Ti2-E microscope equipped with a Yokogawa CSU-W1 spinning disk head, a Hamamatsu ORCA-Flash4.0 camera, NIS-Elements AR (Advanced Research) version 5.02.01 software and a Plan-Apochromat 60x/1.42 Oil DIC objective. Equivalent confocal microscopy or imaging flow cytometry platforms may be used with appropriate optimization of acquisition settings. Our previous study directly compared confocal microscopy and imaging flow cytometry with similar results.^1^

40. Optimize the settings for the confocal microscope.
  a. Mount the unstained, single-stained, negative-control, positive-control, and experimental samples on the microscope.
  b. Use the unstained and single-stained controls to optimize acquisition settings for each fluorescence channel (Table 1).
  c. Verify the absence of bleed-through between channels (Table 1).
41. Establish the Alexa Fluor 647 acquisition range.
  a. Use the negative-control sample to establish the minimum fluorescence intensity so that background fluorescence appears black.
  b. Use the positive-control sample to establish the maximum fluorescence intensity.
    i. Adjust the laser power and detector gain.
    ii. Verify that the antibody staining pattern is clearly distinguishable from the background without saturating the fluorescence signal.

**CRITICAL:** Minimize fluorescence exposure while locating cells to reduce photobleaching. Use the DAPI single-stained control to optimize the DAPI channel so that nuclei are clearly visible without signal saturation.

42. Acquire a predefined number of randomly selected fields for each sample.

**CRITICAL:** Keep laser power, detector gain, exposure time, and all other acquisition settings constant for all experimental samples within the same experiment.

***Note:*** Whenever possible, include the same positive and negative control monoclonal antibodies or serum samples in every imaging experiment to facilitate comparisons between experiments.

**Table 1:** Fluorophores, excitation wavelengths, and detection ranges used for confocal imaging

Staining target	Dye/Fluorophore	Excitation (nm)	Detection range (nm)
Nucleus	DAPI	405	410–480
Autoreactive antibodies	Alexa Fluor 647	640	645–700
CD19	FITC	488	500–555
CD3	PE	561	565–625

### Image analysis

**Timing: 1.5 h for 10 samples (timing depends on the number of samples and fields acquired)**

Analyzing the confocal images confirms that the samples identified as positive via flow cytometry are indeed autoreactive and further clarifies the binding patterns of these antibodies.

43. Open ImageJ (Fiji) and install the custom macro (available at https://github.com/janwisn/Splenocytes) from the plugins menu or use equivalent image analysis software.

**CRITICAL:** Brightness and contrast adjustments should be applied uniformly across all samples within an experiment and used only for visualization.

44. Identify intact splenocytes using DAPI staining and cellular morphology. Exclude dead cells, debris, and imaging artifacts from analysis.
45. Classify antibody binding patterns according to the predominant localization of the Alexa Fluor 647 signal relative to DAPI (e.g., cytoplasmic, nuclear, sub-nuclear, or mixed).
46. Compare staining patterns with the corresponding positive and negative controls.

## Expected outcomes

Successful execution of the FLOWMIST protocol generates complementary flow cytometry and microscopy datasets that enable quantitative screening and validation of antibody binding. Representative results from monoclonal antibody profiling and serum antibody profiling are shown in Figures 2 and 3.

**Figure 2:**
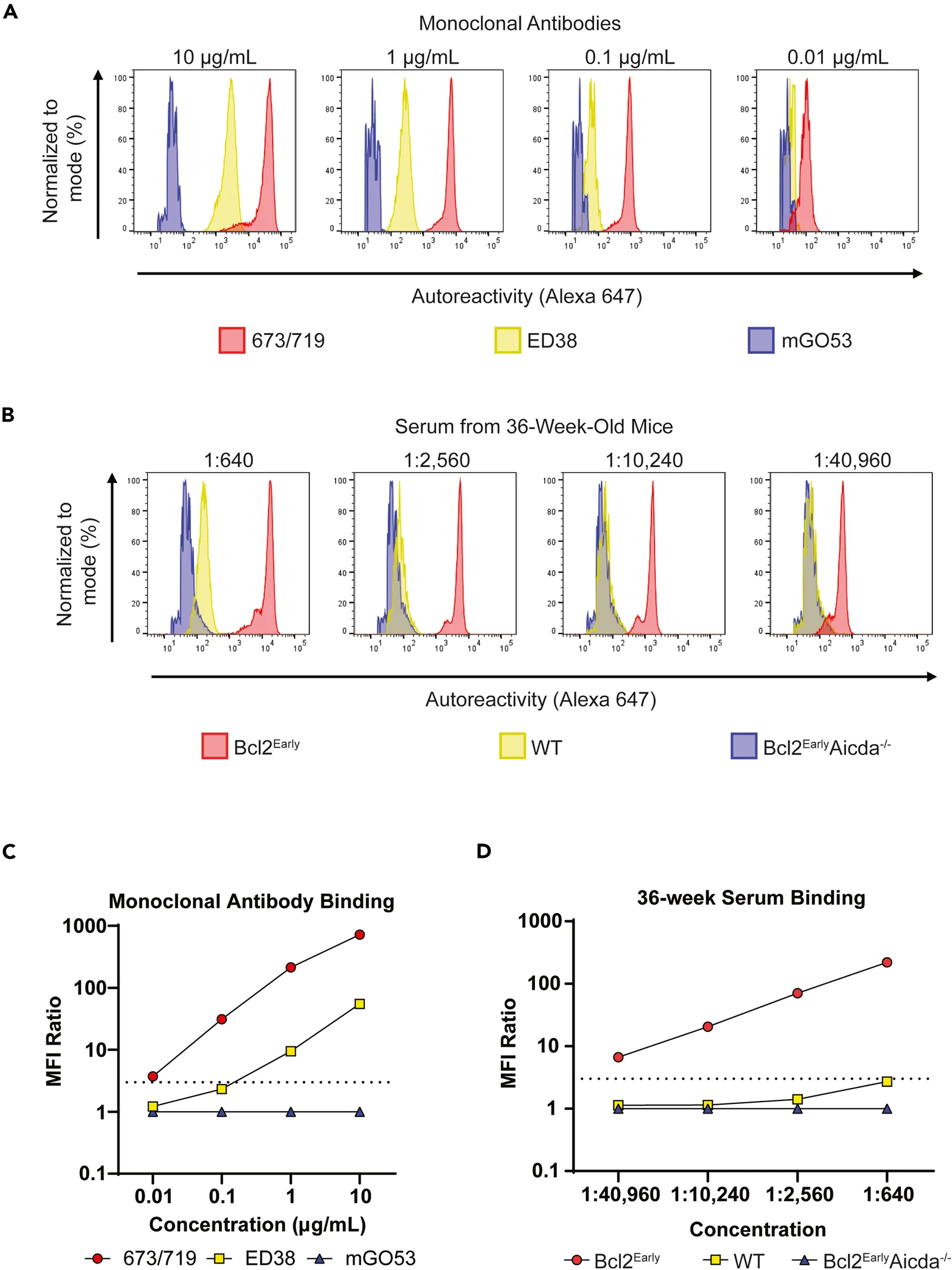
Calculation of MFI ratios following serial dilutions of primary antibody (A and B) Flow cytometry histograms showing the signal intensity of the secondary antibody tested against serial dilutions of the primary antibody. (A) Serial 10-fold dilutions of monoclonal antibodies, beginning at 10 μg/mL and ending at 0.01 μg/mL, which are stained with a secondary antibody conjugated to Alexa Fluor 647 (Alexa647). The peak colors correspond to each monoclonal antibody as follows: red, strongly autoreactive 673/719; yellow, polyreactive ED38; blue, non-reactive mGO53. (B) Serial 4-fold dilutions, beginning at 1:640 and ending at 1:40,960, of mouse serum. The serum was taken from 36-week-old mice and stained with a secondary antibody conjugated to Alexa647 after serial dilutions were performed. The peak colors correspond to each serum type as follows: red, autoreactive Bcl2^Early^; yellow, non-reactive wild-type (WT); blue, non-reactive Bcl2^Early^Aicda^−/−^. (C and D) Plots of the ratio of the secondary antibody Alexa647 MFI signal relative to the non-reactive negative control. Ratios above 3 (dashed line) are considered autoreactive within the context of this experiment, though may vary for individual purposes. (C) Compares Alexa647 MFI ratios between monoclonal antibodies (673/719, autoreactive; ED38, polyreactive) relative to the non-reactive negative control mGO53. (D) Compares Alexa647 MFI ratios between mouse serum samples (Bcl2^Early^, autoreactive; WT, non-reactive) relative to the negative Bcl2^Early^Aicda^−/−^ control.

**Figure 3:**
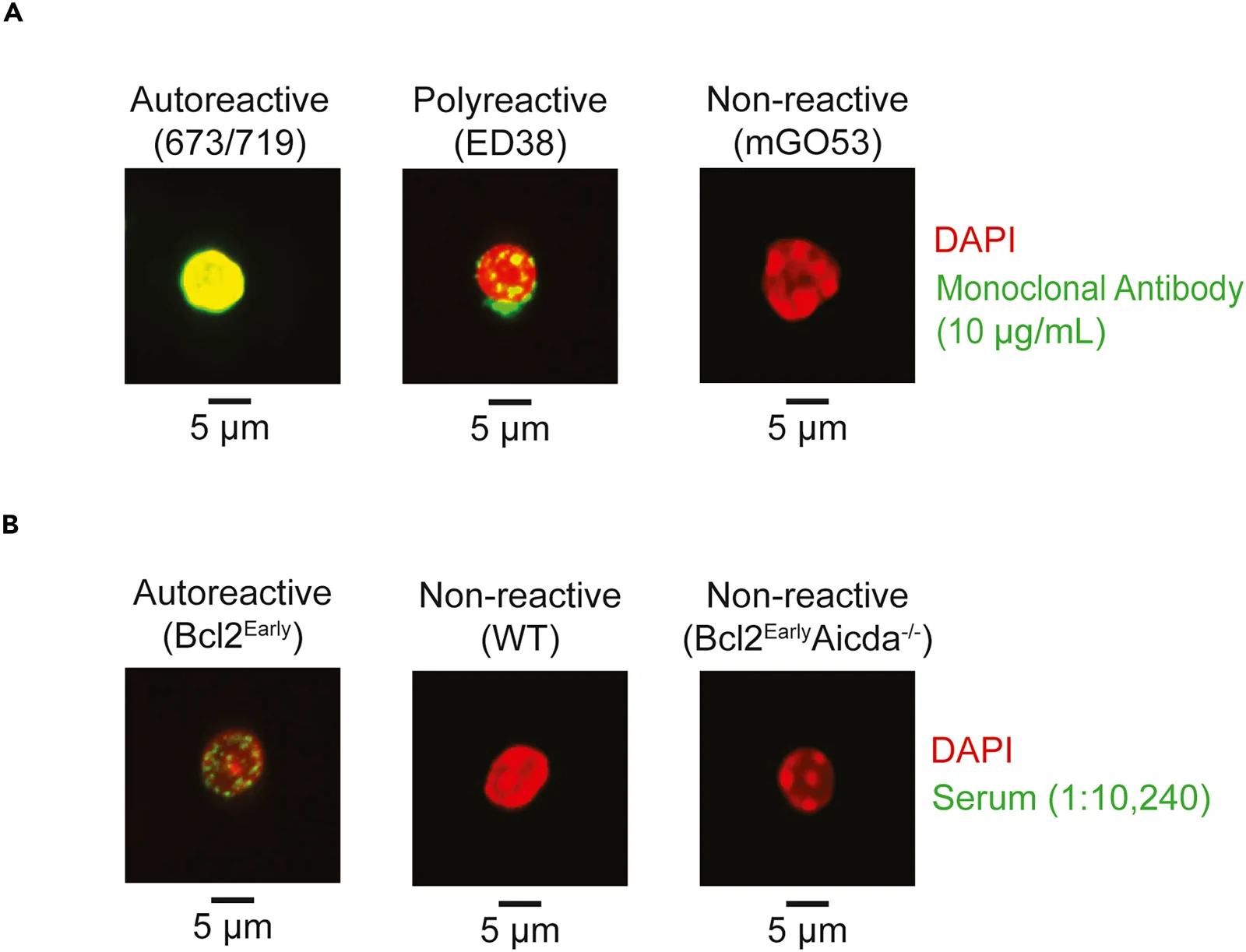
Confocal imaging of optimal flow cytometry samples (A and B) Cropped micrograph of a representative single cell out of images taken at several fields. Confocal scale bars are 5 μm. DAPI is shown in red, the antibody of interest is shown in green, and overlapping regions are shown in yellow. (A) Monoclonal antibody samples of known autoreactivity status (673/719, anti-nuclear; ED38, polyreactive; mGO53, non-reactive) at a concentration of 10 μg/mL were stained with secondary antibody conjugated to Alexa Fluor 647 (Alexa647). (B) Serum samples from 36-week-old mice of different strains (Bcl2^Early^, nuclear speckled autoreactive; WT, non-reactive; Bcl2^Early^Aicda^−/−^, non-reactive) at a 1:10,240 dilution were stained with a secondary antibody conjugated to Alexa647.

For monoclonal antibodies, the magnitude of the fluorescence signal depends on antibody concentration and binding properties. Initial screening may be performed at higher antibody concentrations to maximize sensitivity, whereas serial dilution of positive antibodies enables comparison of relative binding strength. Representative results are shown for the non-reactive control antibody mGO53, the polyreactive control antibody ED38, and the strongly autoreactive control antibody 673/719. mGO53 produces fluorescence comparable to secondary-only controls, whereas ED38 and 673/719 generate concentration-dependent increases in fluorescence intensity (Figure 2). Quantification as the mean fluorescence intensity (MFI) ratio relative to mGO53 provides a convenient metric for comparing antibody binding across samples and concentrations (Figure 2).

For serum profiling, antibody binding similarly depends on serum dilution and antibody titer. We routinely begin screening at a 1:640 serum dilution followed by serial four-fold dilutions to determine endpoint titers. Wild-type serum may produce measurable fluorescence above background, particularly in aged mice, whereas autoreactive serum typically maintains positive staining through higher dilutions. Representative results are shown for serum from wild-type (WT), Mb1^Cre^Rosa26^LSL−Bcl2^ (Bcl2^Early^), and Mb1^Cre^Rosa26^LSL−Bcl2^Aicda^−/−^ (Bcl2^Early^Aicda^−/−^) mice (Figure 2). Serum binding is quantified as the MFI ratio relative to Bcl2^Early^Aicda^−/−^ serum, with values above 3 used to define autoreactivity (Figure 2).

Confocal microscopy validates the flow cytometry results and provides more information regarding antibody binding patterns. After evaluating MFI ratios from flow cytometry, monoclonal antibody and serum samples are imaged with confocal microscopy. This allows for clear differentiation between positive and negative samples while maintaining characteristic staining patterns. For the monoclonal antibodies, samples at the 10 μg/mL concentration were imaged, and negative control mGO53 displayed only DAPI staining, whereas ED38 and 673/719 produced fluorescence that conveyed their cytoplasmic/subnuclear and homogeneous nuclear staining patterns, respectively (Figure 3).^1^ With the serum, samples at 1:10,240 dilutions were imaged. Here, the images for wild-type and Bcl2^Early^Aicda^−/−^ negative controls verified no autoreactivity, and the positive control Bcl2^Early^ serum showed a nuclear speckled staining pattern (Figure 3).

Together, these representative datasets illustrate the dynamic range of the assay and demonstrate how quantitative flow cytometric screening is complemented by microscopic validation of antibody binding patterns. Serial antibody or serum titrations further enable comparison of relative binding strength and endpoint titers.

## Quantification and statistical analysis

Flow cytometry data were analyzed using FlowJo software. The target cell population was gated as described in the protocol, and the mean fluorescence intensity (MFI) of the secondary antibody channel was determined for each sample. For monoclonal antibodies, antibody binding was quantified as the MFI ratio relative to the negative control antibody mGO53 analyzed in parallel. For serum samples, binding was quantified as the MFI ratio relative to Bcl2^Early^Aicda^−/−^ control serum. When serial antibody concentrations or serum dilutions were analyzed, MFI ratios were plotted as a function of concentration or dilution to compare relative antibody binding or determine endpoint titers. Positive samples identified by flow cytometry were subsequently validated by confocal microscopy and classified according to their antibody binding patterns.

When quantitative comparisons between experimental groups are performed, biological and/or technical replicates should be included as appropriate, and statistical analyses should be selected according to the experimental design.

## Limitations

In the workflow described here, fixed and permeabilized target cells are used to detect both surface and intracellular antigens. Fixation and permeabilization may alter protein conformation, disrupt protein complexes, or mask conformational epitopes, potentially reducing or abolishing antibody binding. For analyses limited to surface antigens, fixation and permeabilization may be omitted as described above. In addition, the assay detects only antigens expressed by the selected target cells under the experimental conditions used. Antigens that are absent, expressed at low abundance, or restricted to other cell types or activation states will not be detected. Finally, antibody binding patterns provide information about antigen localization but do not identify the molecular target antigen, which requires complementary approaches such as immunoprecipitation, mass spectrometry, or antigen-specific binding assays.

## Troubleshooting

### Problem 1

Antibody binding differs after changing target cell populations or species (related to “before you begin” and steps 8, 16, and 20).

Antigen expression varies substantially among tissues, cell types, developmental stages, activation states, and species. Consequently, changing the target cells may alter fluorescence intensity or antibody binding patterns.

### Potential solutions

• Target cells: Verify that the selected target cells express the antigen of interest. If necessary, replace the target cells with a more appropriate primary cell population or cell line.
• Assay optimization: Re-optimize antibody concentration or serum dilution after changing target cells. Include the same positive and negative controls used during assay optimization to facilitate comparisons between experiments.
• Secondary detection: Verify compatibility of the secondary antibody with the species and isotype of the primary antibody. Ensure lack of cross-reactivity to optional surface stain antibodies.

### Problem 2

Little or no fluorescence is detected for antibodies expected to be autoreactive (related to “before you begin” and steps 8, 12–16, and 20).

Failure to detect antibody binding may result from low antigen expression in the selected target cells, loss of epitope recognition during fixation or permeabilization, insufficient antibody concentration, or incompatibility of the secondary detection reagent.

### Potential solutions

• Target cell selection: Verify that the selected target cells express the antigen of interest. If necessary, replace the target cells with a more appropriate primary cell population or cell line.
• Fixation and permeabilization: Optimize fixation or permeabilization conditions if the target antigen contains fixation-sensitive epitopes.
• Primary antibody concentration: Increase the antibody concentration or reduce the serum dilution. For unknown samples, initial screening at higher antibody concentrations may improve sensitivity before performing serial titrations.
• Secondary antibody: Confirm that the secondary antibody recognizes the species and isotype of the primary antibody.

### Problem 3

Limited cells remain when acquiring via flow cytometry or imaging (related to Steps 1, 8, 15, 24, and 29).

With the number of staining and washing steps, it is normal to lose cells along the way. Having more cells available for acquisition allows for better analysis of antibody autoreactivity and staining patterns. With this protocol, acquiring around 50,000 cells from the flow cytometry portion of the sample was typical. However, proper analysis may be performed with as few as 10,000 cells depending on the experiment. Samples with lower cell numbers, or too much debris, can produce secondary antibody staining with high background or bimodal distributions (Figure 4). This can make it harder to quantify autoreactivity.

**Figure 4:**
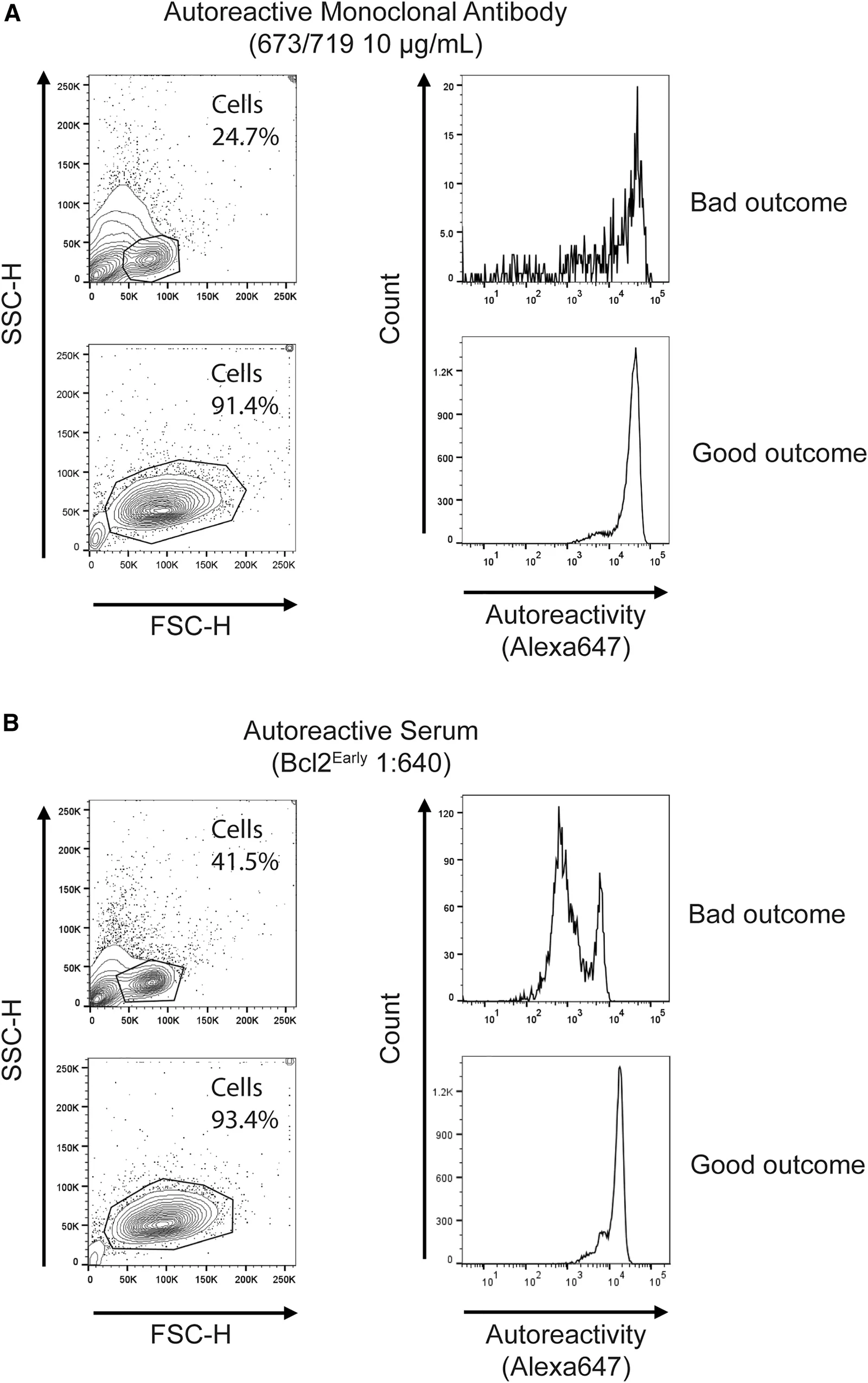
Comparison of bad versus good outcomes (A and B) Flow cytometry plots comparing FSC/SSC plots (left) and autoreactivity histograms (right) of positive control samples across two independent experiments, one done poorly (top) and the other done properly (bottom). (A) Comparisons of good and bad outcomes using the same autoreactive 673/719 monoclonal antibody at a 10 μg/mL concentration. In the bad outcome, there are many dead cells and debris present in the FSC/SSC plot, with only around 1,600 cells able to be gated. As a result, the autoreactivity histogram shows high background, which can negatively impact MFI values used for evaluation. Conversely, a good outcome is shown that contains around 54,000 gated cells and has one clear positive peak with little background. (B) Comparisons of good and bad outcomes using the same autoreactive serum from a 36-week-old Bcl2^Early^ mouse at a 1:640 dilution. A similar problem is observed with a lot of debris and dead cells present, and only around 5,000 cells able to be analyzed. Here, the resulting autoreactivity histogram has a bimodal distribution, possibly due to cells losing their nuclei during wash stages and staining differently. The good outcome, however, contained around 46,000 cells and provided one positive peak demonstrating clear autoreactivity.

### Potential solutions

• Reagent expiration: Ensure that reagents are made fresh for each experiment. Efficacy of expired buffers can degrade, and altered pH or molarity can affect cell survival.
• Washing technique: Flick plates over the sink in one gentle, yet confident, motion to discard the supernatant after each wash step.
• Resuspension: When resuspending, particularly with the Perm/Wash buffer, only gently pipette up and down a few times to avoid excessive shearing stress that lyses cells.
• Minimizing stains: Evaluate if surface staining is necessary for sufficient analysis of autoreactivity, as this is an optional step that adds more washes.
• Separation of flow cytometry and microscopy: Consider acquiring the full sample during flow cytometry and performing an independent experiment for confocal imaging.

### Problem 4

High background fluorescence is observed in negative control samples (related to Steps 5–7, 16, 19–24).

Elevated background fluorescence reduces assay sensitivity and may interfere with discrimination of weakly autoreactive antibodies. High background most commonly results from insufficient blocking, nonspecific secondary antibody binding, or inadequate washing.

### Potential solutions

• Blocking: Ensure adequate Fc receptor blocking before antibody staining.
• Negative controls: Include secondary-only controls together with the non-reactive control antibody mGO53 or wild-type control serum in every experiment.
• Secondary antibody: Reduce the concentration of the secondary antibody if nonspecific staining is observed.
• Washing: Increase the number or volume of wash steps following primary and secondary antibody incubations.

### Problem 5

Loss of antibody binding following fixation and permeabilization (related to Steps 12–15).

Some epitopes are sensitive to fixation or permeabilization, resulting in reduced or absent antibody binding despite expression of the target antigen.

### Potential solutions

• Fixation: Reduce fixation time or optimize fixative concentration if fixation-sensitive epitopes are suspected.
• Permeabilization: Compare alternative permeabilization reagents or detergent concentrations. If only surface antigens are being analyzed by flow cytometry, omit fixation and permeabilization and perform staining in FACS buffer.
• Controls: Validate modified conditions using known positive control antibodies before analyzing experimental samples.

### Problem 6

Weak or inconsistent fluorescence is observed when profiling serum samples (related to Steps 16, 20, and 28).

Serum antibody concentrations and autoreactive antibody titers vary substantially among individual animals and experimental models. Consequently, a single serum dilution may not be optimal for all samples, particularly when comparing different ages, disease models, or experimental conditions.

### Potential solutions

• Serum dilution: Begin screening at the recommended starting dilution and perform serial dilutions to determine endpoint titers.
• Controls: Include wild-type serum and a previously characterized positive serum sample in every experiment.
• Secondary antibody: Confirm compatibility of the secondary antibody with the immunoglobulin isotype being analyzed.
• Replicates: Repeat samples with weak positive signals to confirm reproducibility.

### Problem 7

Confocal microscopy does not reproduce the flow cytometry results (related to Steps 16, and 40–42).

Flow cytometry and confocal microscopy evaluate antibody binding using different readouts. Weakly positive antibodies or suboptimal microscope settings may reduce the visibility of antibody staining.

### Potential solutions

• Signal intensity: Confirm that the antibody produces a reproducible fluorescence shift (MFI ratio > 3) by flow cytometry before microscopy.
• Image acquisition: Optimize laser power, detector gain, and exposure settings while avoiding saturation.
• Representative images: Acquire images from multiple fields of view before classifying antibody binding patterns
• Antibody concentration: If staining remains weak, repeat microscopy using a higher monoclonal antibody concentration or lower serum dilution.

## Resource availability

### Lead contact

Further information and requests for resources and reagents should be directed to and will be fulfilled by the lead contact, Christian T. Mayer (christian.mayer@nih.gov).

### Technical contact

Technical questions on executing this protocol should be directed to and will be answered by the technical contact, Christian T. Mayer (christian.mayer@nih.gov).

### Materials availability

This study did not generate new unique reagents.

### Data and code availability

The ImageJ macro used in this protocol is available at GitHub: https://github.com/janwisn/Splenocytes, with an archived version of record at Zenodo: https://doi.org/10.5281/zenodo.22946432. The published article includes all datasets generated and analyzed during this study.

## Acknowledgments

We thank Jeffrey Chiang and Jie Mu for technical support and Assiatu Crossman, Kheem Bisht, Don Plugge, William Hajjar, and Tengfei Zhang for flow cytometry support. This research was supported by the 10.13039/100030692Intramural Research Program of the 10.13039/100000002National Institutes of Health (NIH; 10.13039/100000054National Cancer Institute ZIA BC 011975). The content of this publication does not necessarily reflect the views or policies of the Department of Health and Human Services nor does mention of trade names, commercial products, or organizations imply endorsement by the US Government. The graphical abstract was created using Biorender.com.

## Author contributions

Conceptualization, C.T.M.; methodology, C.T.M., M.I., U.A., and J.W.; investigation, M.I. and U.A.; writing – original draft, C.T.M., M.I., U.A., and J.W.; writing – review and editing, C.T.M., M.I., U.A., and J.W.; funding acquisition, C.T.M.; supervision, C.T.M.

## Declaration of interests

The authors declare no competing interests.

## Declaration of generative AI and AI-assisted technologies in the manuscript preparation process

During the preparation of this work, the authors used ChatGPT provided by the US Department of Health and Human Services in order to optimize language. After using these tools, the authors reviewed and edited the content as needed and take full responsibility for the content of the publication.
